# Optimization of HIV drugs through MCDM technique Analytic Hierarchy Process(AHP)

**DOI:** 10.1371/journal.pone.0316617

**Published:** 2025-01-17

**Authors:** Fozia Bashir Farooq, Sobia Sultana, Nouf Abdulrahman Alqahtani, Muhammad Imran

**Affiliations:** 1 Department of Mathematics and Statistics, College of Science, Imam Mohammad Ibn Saud Islamic University (IMSIU), Riyadh, Saudi Arabia; 2 Prince Mohammad Bin Fahd University, Al Khobar, Saudi Arabia; Federal University of ABC, BRAZIL

## Abstract

Topological indices are crucial tools for predicting the physicochemical and biological features of different drugs. They are numerical values obtained from the structure of chemical molecules. These indices, particularly the degree-based TIs are a useful tools for evaluating the connection between a compound’s structure and its attributes. This study addresses the research problemof how to optimize drug design for HIV treatment using degree-based topological indices. The need for safer and more effective medicines for HIV is further emphasized by the advent of drug resistance and severe negative effects from current therapies. Employing degree-based graph invariants, the study investigates 13 HIV drugs by applying a quantitative structure-property relationship (QSPR) technique to associate their molecular structures with their physical properties. HIV drugs are ranked using the Analytic Hierarchy Process (AHP) according to specific parameters. The findings of the study demonstrate how well these approaches can determine the most effective possible drug combinations and designs, offering insightful information in developing improved HIV treatments.

## 1. Introduction

Infectious diseases have been always a constant threat to individuals globally. Contagious infections can be transmitted from animals to humans, humans to humans or humans to animals. The human immunodeficiency virus (HIV), responsible for causing acquired immune deficiency syndrome (AIDS), was discovered by French researchers forty years ago [1], following the initial reports of AIDS by American scientists two years earlier [2]. Since the onset of the HIV/AIDS pandemic, it has reached every country worldwide, leading to over 84 million infections and more than 40 million deaths [3]. HIV is a human disease that affects millions of people and impairs the body’s ability to fight off common infections. The immune system, which is composed of white blood cells, antibodies, and other cells, is the body’s defence mechanism. In vitro tests demonstrate that HIV spreads through two mechanisms: virus-to-cell (VTC) and cell-to-cell (CTC White blood cells called CD4 guard off attackers, but HIV targets and infiltrates CD4 cells). When HIV binds to a CD4 cell and inserts its proteins and genetic material into it, the HIV life cycle starts. Following that, HIV RNA is transformed into HIV DNA by an enzyme known as reverse transcriptase and HIV DNA is integrated into the host CD4 cell’s DNA by an enzyme known as integrase. The generated HIV material is then processed by the protease enzyme so that it can be assembled into an immature virus. Eventually, as the cycle repeats itself, mature HIV components are released into the body [4]. There is no cure for HIV but antiretroviral medications or ARVs reduce HIV reproduction by interrupting the HIV life cycle [5]. People generally take a combination of medications called highly active antiretroviral therapy(HAART) or combination antiretroviral therapy(CART) as the mechanism of action of drugs [6, 7] while nucleoside reverse transcriptase inhibitors (NRTI) and non-nucleoside reverse transcriptase inhibitors (NNRTI) block the reverse transcriptase enzymes from turning HIV RNA into HIV DNA, entry inhibitors prohibit HIV material from entering CD4 cells. Subsequently, protease inhibitors prevent the generation of new HIV material by protease enzymes, whereas integrase inhibitors prevent HIV integrase enzymes from integrating HIV DNA into CD4 DNA. We add other drugs like 1 NNRTI, 1 PI or 1 INI(boosted) with backbone therapy that is 2 NRTI in antiviral therapy according to CDC guidelines [8]. HIV transmission through immediate cell-to-cell contact appears to be more effective and potent compared to virus-to-cell transmission [9].

A topological index is a graph-invariant number that is calculated from a molecular graph. Topological indices, used in chemical graph theory, are numerical descriptors that encapsulate the structural information of molecules through graph-based representations, with atoms as nodes and bonds as edges [10]. Quantitative Structure-Property Relationship QSPR is a technique used in materials science and chemistry to predict a molecule’s characteristics based on its chemical structure. In this study, we shall consider the degree-based topological indices for HIV medications. Topological indices play an important role in predicting the impact of various chemical compounds used in different fields such as chemistry, bioinformatics and drug design [11]. They allow scientists to analyze and improve molecular structures to boost drug effectiveness and bioavailability. Scientists are testing research-based medicinal remedies as potential medications. These indices are used in predicting a range of chemical and biological properties [12] through QSPR and quantitative structure-activity relationship (QSAR) research [13]. Recent studies emphasize their significance in drug design, materials science, and environmental chemistry, demonstrating their enduring importance in understanding the connections between molecular structure and properties which means QSPR models help to determine the optimal relationship between topological indices (TIs) and physical properties.

This work aims to examine the different components and prioritize the best medication to prevent HIV. Many studies have identified a strong correlation between molecular structure and various properties of medications and chemicals, including their boiling and melting points. The connection between HIV disease and topological indices lies in their potential to understand the molecular basis of medications used in HIV treatments. These indices enable the analysis and prediction of molecular properties and behaviours, primarily based on their topological structure. Optimizing molecular structures improves pharmacological efficacy and bioavailability [14]. Quantitative Structure-Property Relationship (QSPR) analysis is instrumental in comprehending HIV disease, as it correlates the structural attributes of medications with their properties or effects.

Multi-criteria decision-making (MCDM) techniques are valuable area in OR that are utilized to assess and rank multiple criteria or properties when evaluating the potential therapeutic abilities of chemical compounds [15, 16]. Consequently, integrating MCDM into QSPR analysis strengthens the reliability of molecular evaluations, providing valuable insights for drug discovery and environmental research. This work explores the combination of QSPR analysis and MCDM approaches such as the analytic hierarchy process (AHP) with Operational Research to improve biochemical sciences research. Here, the AHP technique is applied in HIV drug optimization to rank the relative importance of those factors gathered due to its gradually growing applicability. Analytic Hierarchy Process (AHP) was first presented by Saaty in the late 1970s [17, 18]. TIs and pharmacological properties have a significant correlation, as shown by Nasir et al. QSPR modeling of cardiovascular medications [13]. Novel topological properties of chemical structures have found applications in the treatment of COVID-19 [15]. Numerous QSPR analyses have been carried out, each focusing on a different field of pharmaceutical research. Another study uses M-polynomial formulations to investigate degree-based topological indices for anti-HIV medications [19], regression models, and degree-based topological indexes for thirteen HIV/AIDS therapy medications [20]. Furthermore, a QSPR model based on several Revan indices is used to anticipate the physicochemical and pharmacokinetic characteristics of anti-flaviviral medications, such as ADMET (Absorption, Distribution, Metabolism, Excretion, and Toxicity) [21, 22]. Several degree-based topological indices for diabetes medications, including Acarbose, Tolazamide, Miglitol, Prandin, Metformin, Glimepiride, Linagliptin, Pioglitazone, Bromocriptine, and Alogliptin, are studied through the use of QSPR analysis [23]. Finally, without the necessity for experimental trials, statistical analysis is used to predict the characteristics of antiviral medications [24].

Different approaches are used in different decision-making situations to deal with intricate multi-criteria problems. Analytic Hierarchy Process, or AHP, is one such technique that was created to deal with challenging real-world circumstances [25]. The VIKOR approach is a strategy for making multi-criteria decisions (MCDM) or doing decision analyses. This strategy was designed with the premise that, in instances when judgments must be made based on competing and incommensurable requirements, compromise is ideal for conflict resolution. Because the decision-maker prefers the outcome that is closest to the ideal, the alternatives are evaluated using all predetermined criteria. After considering the options, VIKOR chooses the compromise option that is closest to the ideal. Po Lung Yu introduced VIKORE into MCDM in 1973 [26].

Simple Additive Weighting method (SAW), Technique for Order of Preference by Similarities to Ideal Solution (TOPSIS), and Analytic Hierarchy Process(AHP). A decision support system using the Simple Additive Weighting (SAW) MCDM approach is advised to help in laptop selection [27]. Schmidt et al provided insight into the use of AHP in healthcare [28]. According to Triantaphyllou and Mann, AHP is a decision-aid tool that can handle complex decision-making challenges [29]. The AHP seems to be a viable support tool for therapy and treatment selection and evaluation, patient-doctor collaborative decision-making, and the assessment of healthcare technologies and policies [25]. Ehali et al [30] analyse the factors which may influence the decision in the selection of a pharmaceutical product and rank them using the AHP method.

Our inspiration to work on the current study issue stemmed from works on the application of multi-criteria decision-making for the ranking of medications used to treat HIV and an MCDM model design for HER2+ breast cancer treatment procedure utilizing the AHP method [16, 31].

The forthcoming content of this article proceeds as follows: Some basic topics in graph theory an introduction to the degree-based topological indices and a description of HIV drugs relevant to our investigation are provided in section 2. This highlights the importance of looking into the information and the upcoming study. The results and related discussions are covered in Section 3. First, we elaborate on the Steps for performing AHP. Then, alternatives and weight criteria are determined. Furthermore, evaluation is done to ensure the consistency of weight criteria also correlation values are used to derive beneficial and non-beneficial criteria from the QSPR analysis that affect boiling point, flash point, and Complexity. Rankings for boiling, flash point, and complexity are also provided, and weights are distributed according to correlation coefficients. Further discussion, closing thoughts, and recommendations for future research are provided in Section 4.

## 2. Methodology and materials

Chemical graph theory is a blend of chemistry and mathematics. It uses graph theory ideas and rules to represent molecular structures as graphs, where atoms are represented as vertices while chemical bonds as edges. The analysis of molecule structure, connectivity, and characteristics is facilitated by this method [32, 33]. Graph-theoretical descriptors for drug discovery and materials research are being explored, and graph neural networks are being used to predict chemical properties [32, 34]. The drug’s structure is illustrated in this study as a graph ℳ = (V (ℳ), E(ℳ)) where each edge E(ℳ) denotes the chemical bond between these atoms and each vertex V(ℳ) expresses an atom. Every graph is considered connected and simple. A vertex’s degree is determined by the number of connected edges [35]. The notation ℜ(*p*) and ℜ(*q*), respectively, indicate the degree of vertices u and v. In this study, we considered the following indices for chemical graph ℳ, which are defined below:

### Definition 2.1

The following is the definition of the Randic´ index, which was presented by Milan Randic´ in [36]: The sum of reciprocals of the square root of the product of vertex degrees of all edges in the graph. It has the following mathematical representation:

$$R(M)=\sum_{pq\in E(M)}\frac{1}{\sqrt{ℜ(p)\times ℜ(q))}}$$ (1)

### Definition 2.2

The first Zagreb index (referred to as *M*_1_(ℳ)) was introduced and defined by Gutman and Trinajstic´ as the sum of the vertex degrees for each edge in the graph, and the second Zagreb index (referred to as *M*_2_(ℳ)) as the sum of the products of the vertex degrees for each edge in the graph in [37–39]. It has the following mathematical representation:

$$M_{1}(M)=\sum_{pq\in E(M)}\left(ℜ(p)+ℜ(q)\right)$$ (2)

$$M_{2}(M)=\sum_{pq\in E(M)}\left(ℜ(p)\times ℜ(q)\right)$$ (3)

### Definition 2.3

The Hyper Zagreb index was first presented by Shirdel G. H. et al. in [40] and is defined as the total square of the vertex degrees for each edge in the molecular graph ℳ. It has the following mathematical representation:

$$HM(M)=\sum_{pq\in E(M)}{\left[ℜ(p)\times ℜ(q)\right]}^{2}$$ (4)

### Definition 2.4

Fajtlowicz proposed the Harmonic index [41]. It is computed using the harmonic mean of the degrees of the graph’s neighboring vertices. The following is the formula for a graph ℳ ’s harmonic index:

$$H(M)=\sum_{pq\in E(M)}\frac{2}{ℜ(p)+ℜ(q)}$$ (5)

### Definition 2.5

The geometric arithmetic index was proposed by Vukicevic et al. [42] and is defined as the geometric mean plus the arithmetic mean of the degrees of adjacent vertices in the graph. The following is the formula for a molecular graph ℳ's geometric arithmetic index:

$$\text{GA}(M)=\sum_{pq\in \text{E}(M)}\frac{2\sqrt{ℜ(\text{p})\times ℜ(\text{q})}}{ℜ(\text{p})+ℜ(\text{q})}$$ (6)

### Definition 2.6

The forgotten topological index was first described by Furtula et al. [43]. It is the sum of squares of a graph’s vertex degrees. It has the following mathematical representation:

$$F(M)=\sum_{pq\in E(M)}\left[{\left(ℜ(p)\right)}^{2}+{\left(ℜ(q)\right)}^{2}\right]$$ (7)

### Definition 2.7

The Sum-connectivity Index, or SCI(ℳ), was first introduced by Zhou et al. [44]. It is a measurement of the sum of the reciprocal of square roots of the sums of degrees of nearby vertices in the network. The following is the formula for a molecular graph ℳ's Sum-Connectivity Index:

$$\mathrm{SCI}(M)=\sum_{pq\in E(G)}\frac{1}{\sqrt{ℜ(p)+ℜ(q)}}$$ (8)

### Definition 2.8

Estrada presented and examined the Atom-bond connectivity index (abbreviated as ABC(ℳ)) in [45]. It has the following definition:

$$\mathrm{ABC}(M)=\sum_{pq\in \text{E}(M)}\frac{\sqrt{ℜ(\text{p})+ℜ(\text{q})-2}}{ℜ(\text{p})\times ℜ(\text{q})}$$ (9)

Tipranavir, a sulfonamide-containing dyhydropyrone and nonpeptidic protease inhibitor targets the HIV protease. Ritonavir and tripranavir are given together to treat HIV. The chemical formula for Tipranavir is C_31_H_33_ F_3_N_2_ O_5_S. It is used to prevent the formation of mature virions in HIV-1 treatment. Lamivudine also known as 3TC with chemical formula C_8_H_11_ N_3_ O_3_S, is an antiviral drug used to treat and prevent HIV/AIDS. In cases where alternative treatments are not feasible, it is also used to treat chronic hepatitis B. It is successful in combating both HIV-1 and HIV-2. Nelfinavir, an antiretroviral drug used to treat HIV/AIDS, with the chemical formula C_32_H_45_N_3_ O_4_S. As a member of the protease inhibitor (PI) class of medications, nelfinavir is almost always used in conjunction with other antiretroviral medications.

Maraviroc has the molecular formula C_29_H_41_ F_2_N_5_ O_4_. Maraviroc in conjunction with other antiretroviral drugs treats only CCR5-tropic HIV-1 infection. Maraviroc is a negative allosteric modulator and entrance inhibitor of the CCR5 receptor, which is present on the surface of some human cells. For the majority of HIV strains, the chemokine receptor CCR5 is a crucial co-receptor and is required for the virus’s entrance into the host cell. By binding to CCR5, the medication prevents the HIV protein gp120 from binding to the receptor. After that, HIV is unable to infiltrate human T cells and macrophages. Emtricitabine with molecular formula C_8_H_10_FN_3_O_3_S, also referred to as FTC, is a nucleoside reverse transcriptase inhibitor (NRTI) used in the therapy of HIV infection for both adults and pediatric. When combined with emtricitabine (FTC), tenofovir disoproxil fumarate (TDF) or tenofovir alafenamide are administered. Cytidine’s counterpart is emtricitabine. The drug inhibits HIV reverse transcriptase, which stops HIV RNA from being translated into DNA. Etravirine with molecular formula C_20_H_15_BrN_6_O is a non-nucleoside reverse transcriptase inhibitor (NNRTI). Unlike other agents in its class, resistance to other NNRTIs does not appear to cause resistance to Etravirine.

S1 Fig in S1 File provides chemical structures of thirteen medications for HIV illness, including Vidaza, Lamivudine, Darunavir, Disovey, Maraviroc, Tenofovir, Tipranavir, Atazanavir, Lopivirine, Abacavir, Nelfnavir, and Treforant. The research highlights three physical and structural features of the thirteen medications used to treat HIV, with a focus on complexity, flash point, and boiling point (BP), using an MCDM approach for ranking based on QSPR analysis. Table 1 [34] lists the Values of Topological Indices for drugs that are utilized in QSPR modeling. Correlation Coefficients for topological indices provided in Table 2 are taken from [34]. Regression analysis is performed using the linear model.

**Table 1:** Decision matrix(Values of Topological Indices for drug candidates).

Drug	ABC(ℳ)	GA(ℳ)	F(ℳ)	H(ℳ)	HM(ℳ)	M1(ℳ)	M2(ℳ)	R(ℳ)	S(ℳ)
*Vidaza*	12.96	17.265	234	7.63	444	88	105	8.04	8.25
Lamivudine	11.45	15.49	194	6.93	370	76	88	7.20	7.42
Darunavir	25.46	33.33	467	14.86	867	171	200	15.72	16.13
Disovey	12.23	16.36	214	7.27	406	82	96	7.61	7.83
Maraviroc	29.42	39.63	538	17.03	1018	202	240	17.72	18.71
Tenofovir	26.37	34.29	444	15.60	822	170	189	16.47	16.83
Tripranavir	32.55	42.93	630	18.84	1160	224	265	19.83	20.72
Atazanavir	38.69	50.51	676	22.74	1250	254	287	24.01	24.46
Lopinavir	34.46	46.66	558	21.0	1068	224	255	21.71	22.39
Abacavir	16.92	23.51	306	10.0	592	118	143	10.24	10.92
Etravirine	20.29	26.87	356	11.73	672	136	158	12.33	12.79
Nelfinavir	32.05	41.92	604	18.28	1114	218	255	19.30	19.98
Toreforant	26.86	35.55	458	15.90	858	176	200	16.65	17.11

**Table 2:** Correlation coefficients for topological indices for physicochemical properties.

Topological Index	Boiling point	Flash point	Complexity
**ABC(**ℳ**)**	0.865	0.864	0.926
**GA(**ℳ**)**	0.882	0.882	0.919
**F(**ℳ**)**	0.813	0.813	0.923
**H(**ℳ**)**	0.877	0.877	0.923
**HM(**ℳ**)**	0.835	0.835	0.922
**M1(**ℳ**)**	0.860	0.860	0.924
**M2(**ℳ**)**	0.859	0.858	0.919
**RA(**ℳ**)**	0.868	0.868	0.986
**S(**ℳ**)**	0.876	0.876	0.923

## 3. Results and discussion

### 3.1 Steps for performing Analytic Hierarchy Process(AHP)

AHP consists of three parts. The ultimate goal of the problem with all possible solutions, known as alternatives, and the criteria that will be used to judge the alternatives.

Level 1 is the goal, which is to select the best Alternative(result) given. The second level contains the selection criteria, however, alternatives are put forward at the third level

The next step is to conduct pairwise comparisons among the selected criteria. The scale takes place from one to nine, with one indicating that the two elements are the same or equally important. The assignment of hierarchy is shown in Table 3 below.

**Table 3:** Fundamentals of scale.

Relative Importance Intensity	The Importance Scale Definition
1	The goal is equally aided by both criteria.
3	Moderate Priority of one criteria over the other.
5	Strong Priority of one criteria over the other.
7	Very strong Priority of one criteria over the other.
9	Extreme strong Priority of one criteria over the other.
2,4,6,8	values that are moderate between the two adjustment assessments

We construct pairwise comparison matrix M based on the previous step’s assessments. The value *m*_*ij*_ for the position (*i*,*j*) of the pairwise comparison matrix M has been determined by using scales provided by Table 3 in addition to the following rule.


$$m_{ij}\geq 0,\;m_{ji}=\frac{1}{m_{ij}},\;m_{ii}=1$$

**Fig 1:**
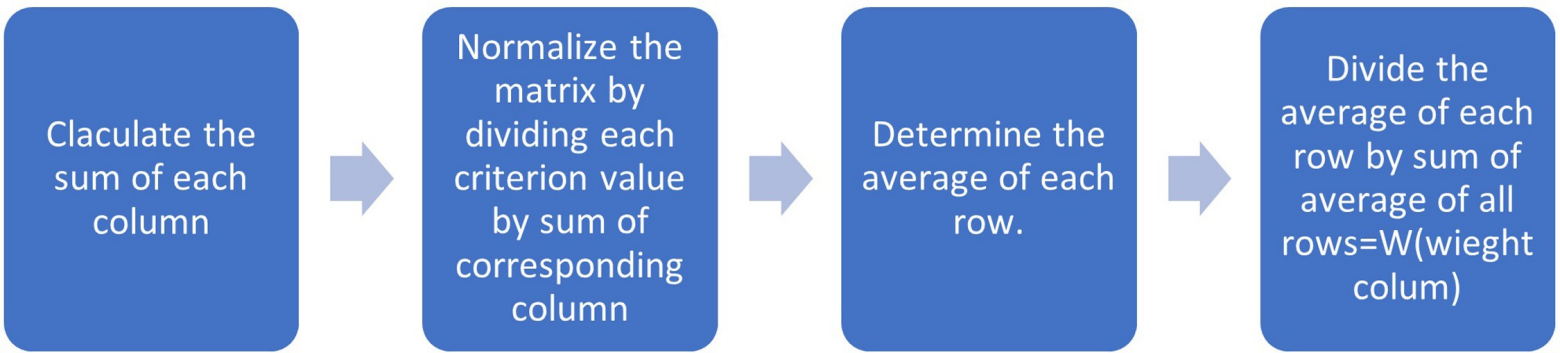
Weight determination steps.

To determine the weight assigned to each criterion, the guidelines given in Fig 1 are employed.

#### 3.1.1 Consistency check

This step is crucial as it ensures that we have not performed any irrational comparison and that the criteria weights are consistent before moving to the next step. To achieve the consistency of this method following steps are required to perform.

- Calculate the vector *W_s_* = *M* * *W*
- Calculate Consistency vector $V_{c}=W_{s}*\frac{1}{W}$
- *λ_max_* *= average value of V*_*c*_
- $Consistency\;Index\;(C.I)=\frac{\lambda_{max}-l}{l-1}$, where *l* is the number of criteria.
- $Consistency\;Ratio\;C.R=\frac{C.I}{\;Random\;Index\;}$, Radom Index Table was provided by Satty [17]

**Table:** 

1	2	3	4	5	6	7	8	9	10	11	12	13
0.00	0.00	0.58	0.90	1.12	1.24	1.32	1.41	1.45	1.49	1.51	1.48	1.56

- If *C*.*R* < 0.1: then the assessments can be considered meaningful and the Matrix is consistent. We can move to the next step.
- *Rank colum R =* Normalized Decision Matrix * W

### 3.2 Assessment of AHP in conjunction with boiling point(BP) QSPR extractions

In the scenario, outcomes are extracted from QSPR analysis based on correlation values.

**Fig 2:**
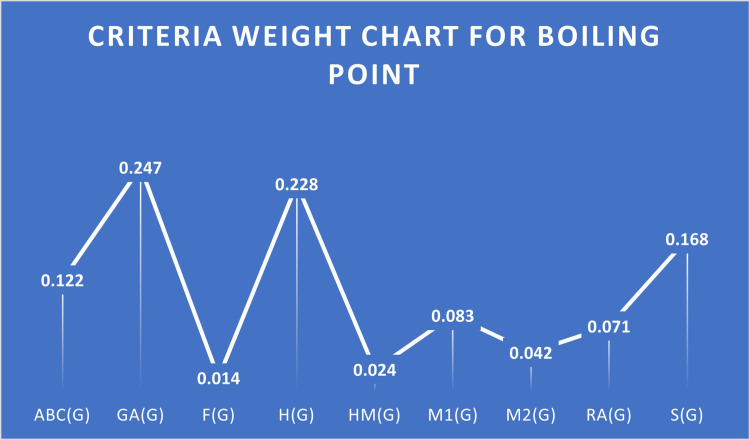
Criteria weight chart for boiling point.

A higher correlation among some criteria suggests that this criterion is beneficial. S1 Table in S1 File lists the criteria that are beneficial and non-beneficial. The decision matrix will be normalized using beneficial criteria with the linear max technique. A pairwise Comparison of criteria under the Boiling point is given in Table 4, Normalized Decision Matrix for Flash Point Case is given in Table 5, and Ranks of HIV drugs for Boiling Point Case are given in Table 6. Fig 2 provides the criteria weight chart for the Boiling point. Microsoft Excel is used to analyze the data,as well as to create graphs for visualization.

**Table 4:** Pairwise comparison matrix M for boiling point case.

	ABC(ℳ)	GA(ℳ)	F(ℳ)	H(ℳ)	HM(ℳ)	M1(ℳ)	M2(ℳ)	RA(ℳ)	S(ℳ)
**ABC(**ℳ**)**	1.000	0.333	7.000	0.333	5.000	4.000	3.000	4.000	0.333
**GA(**ℳ**)**	3.000	1.000	9.000	2.000	7.000	3.000	5.000	3.000	2.000
**F(**ℳ**)**	0.143	0.111	1.000	0.111	0.333	0.143	0.200	0.143	0.111
**H(**ℳ**)**	3.000	0.500	9.000	1.000	7.000	3.000	5.000	3.000	4.000
**HM(**ℳ**)**	0.200	0.143	3.000	0.143	1.000	0.200	0.333	0.200	0.143
**M1(**ℳ**)**	0.250	0.333	7.000	0.333	5.000	1.000	3.000	2.000	0.333
**M2(**ℳ**)**	0.333	0.200	5.000	0.200	3.000	0.333	1.000	0.333	0.200
**RA(**ℳ**)**	0.250	0.333	7.000	0.333	5.000	0.500	3.000	1.000	0.333
**S(**ℳ**)**	3.000	0.500	9.000	0.250	7.000	3.000	5.000	3.000	1.000
**Weights**	0.122	0.247	0.014	0.228	0.024	0.083	0.042	0.071	0.168

The Consistency Ratio for these weights is 0.080618 < 0.1 which means weights are consistent.

**Table 5:** Normalized decision matrix NM (BP Case).

Drug	ABC (ℳ)	GA(ℳ)	F (ℳ)	H (ℳ)	HM(ℳ)	M1(ℳ)	M2 (ℳ)	R(ℳ)	S(ℳ)
*Vidaza*	1.132	0.342	1.206	0.336	1.200	1.158	1.193	0.335	0.337
Lamivudine	1.000	0.307	1.000	0.305	1.000	1.000	1.000	0.300	0.303
Darunavir	2.224	0.660	2.407	0.653	2.343	2.250	2.273	0.655	0.659
Disovey	1.068	0.324	1.103	0.320	1.097	1.079	1.091	0.317	0.320
Maraviroc	2.569	0.785	2.773	0.749	2.751	2.658	2.727	0.738	0.765
Tenofovir	2.303	0.679	2.289	0.686	2.222	2.237	2.148	0.686	0.688
Tripranavir	2.843	0.850	3.247	0.828	3.135	2.947	3.011	0.826	0.847
Atazanavir	3.379	1.000	3.485	1.000	3.378	3.342	3.261	1.000	1.000
Lopinavir	3.010	0.924	2.876	0.923	2.886	2.947	2.898	0.904	0.915
Abacavir	1.478	0.465	1.577	0.440	1.600	1.553	1.625	0.426	0.446
Etravirine	1.772	0.532	1.835	0.516	1.816	1.789	1.795	0.514	0.523
Nelfinavir	2.799	0.830	3.113	0.804	3.011	2.868	2.898	0.804	0.817
Toreforant	2.346	0.704	2.361	0.699	2.319	2.316	2.273	0.693	0.700
**Weights**	**0.122**	**0.247**	**0.014**	**0.228**	**0.024**	**0.083**	**0.042**	**0.071**	**0.168**

**Table 6:** Ranks Of HIV drugs for boiling point case.

Drugs	*R*	RANK
***Vidaza***	0.572558	11
**Lamivudine**	0.503511	13
**Darunavir**	1.115009	8
**Disovey**	0.537747	12
**Maraviroc**	1.301645	5
**Tenofovir**	1.132908	7
**Tripranavir**	1.44147	3
**Atazanavir**	1.673944	1
**Lopinavir**	1.502785	2
**Abacavir**	0.759968	10
**Etravirine**	0.884555	9
**Nelfinavir**	1.402668	4
**Toreforant**	1.164971	6

### 3.3 Assessment of AHP in method with flash point QSPR extractions

**Fig 3:**
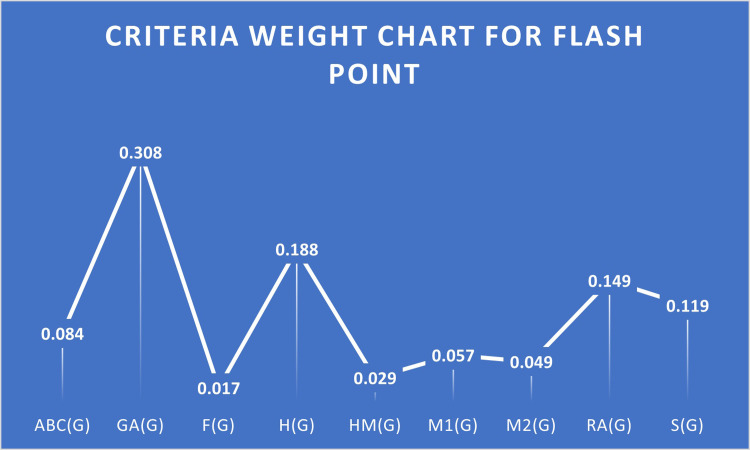
Criteria weight chart for flash point.

In the scenario, outcomes are extracted from QSPR analysis based on correlation values under the study of flash points. A higher correlation among some criteria suggests that this criterion is beneficial. S2 Table in S1 File lists the criteria that are beneficial and non-beneficial. Pairwise Comparison of criteria under flash point is given in Table 7, Normalized Decision Matrix for flash Point Case is given in Table 8, and Ranks of HIV drugs for Flash Point Case are given in Table 9. Fig 3 provides the criteria weight chart for flash points.

**Table 7:** Pairwise comparison matrix for flash point case.

	ABC(ℳ)	GA(ℳ)	F(ℳ)	H(ℳ)	HM(ℳ)	M1(ℳ)	M2(ℳ)	RA(ℳ)	S(ℳ)
**ABC(**ℳ**)**	1.000	0.200	5.000	0.333	3.000	4.000	4.000	0.333	0.333
**GA(**ℳ**)**	5.000	1.000	9.000	3.000	7.000	5.000	5.000	3.000	3.000
**F(**ℳ**)**	0.200	0.111	1.000	0.143	0.333	0.200	0.200	0.143	0.143
**H(**ℳ**)**	3.000	0.333	7.000	1.000	5.000	3.000	3.000	4.000	2.000
**HM(**ℳ**)**	0.333	0.143	3.000	0.200	1.000	0.333	0.333	0.200	0.200
**M1(**ℳ**)**	0.250	0.200	5.000	0.333	3.000	1.000	2.000	0.333	0.333
**M2(**ℳ**)**	0.250	0.200	5.000	0.333	3.000	0.500	1.000	0.333	0.333
**RA(**ℳ**)**	3.000	0.333	7.000	0.250	5.000	3.000	3.000	1.000	4.000
**S(**ℳ**)**	3.000	0.333	7.000	0.500	5.000	3.000	3.000	0.250	1.000
**Weights**	0.084	0.308	0.017	0.188	0.029	0.057	0.049	0.149	0.119

The Consistency Ratio for these weights is 0.090909 < 0.1 that means weights are consistent.

**Table 8:** Normalized decision matrix for flash point case.

*DRUG*	ABC(ℳ)	GA(ℳ)	F(ℳ)	H(ℳ)	HM(ℳ)	M1(ℳ)	M2(ℳ)	RA(ℳ)	S(ℳ)
***Vidaza***	1.132	0.342	1.206	0.336	1.200	1.158	1.193	0.335	0.337
**Lamivudine**	1.000	0.307	1.000	0.305	1.000	1.000	1.000	0.300	0.303
**Darunavir**	2.224	0.660	2.407	0.653	2.343	2.250	2.273	0.655	0.659
**Disovey**	1.068	0.324	1.103	0.320	1.097	1.079	1.091	0.317	0.320
**Maraviroc**	2.569	0.785	2.773	0.749	2.751	2.658	2.727	0.738	0.765
**Tenofovir**	2.303	0.679	2.289	0.686	2.222	2.237	2.148	0.686	0.688
**Tripranavir**	2.843	0.850	3.247	0.828	3.135	2.947	3.011	0.826	0.847
**Atazanavir**	3.379	1.000	3.485	1.000	3.378	3.342	3.261	1.000	1.000
**Lopinavir**	3.010	0.924	2.876	0.923	2.886	2.947	2.898	0.904	0.915
**Abacavir**	1.478	0.465	1.577	0.440	1.600	1.553	1.625	0.426	0.446
**Etravirine**	1.772	0.532	1.835	0.516	1.816	1.789	1.795	0.514	0.523
**Nelfinavir**	2.799	0.830	3.113	0.804	3.011	2.868	2.898	0.804	0.817
**Toreforant**	2.346	0.704	2.361	0.699	2.319	2.316	2.273	0.693	0.700

**Table 9:** Ranks Of HIV drugs for flash point case.

*DRUG*	*R*	RANK
***Vidaza***	0.533203	11
**Lamivudine**	0.468507	13
**Darunavir**	1.037305	8
**Disovey**	0.500405	12
**Maraviroc**	1.211249	5
**Tenofovir**	1.05162	7
**Tripranavir**	1.341732	3
**Atazanavir**	1.55525	1
**Lopinavir**	1.39715	2
**Abacavir**	0.708131	10
**Etravirine**	0.822209	9
**Nelfinavir**	1.304452	4
**Toreforant**	1.082531	6

### 3.4 Assessment of AHP in conjunction with complexity QSPR extractions

In the scenario, outcomes are extracted from QSPR analysis based on correlation values.

**Fig 4:**
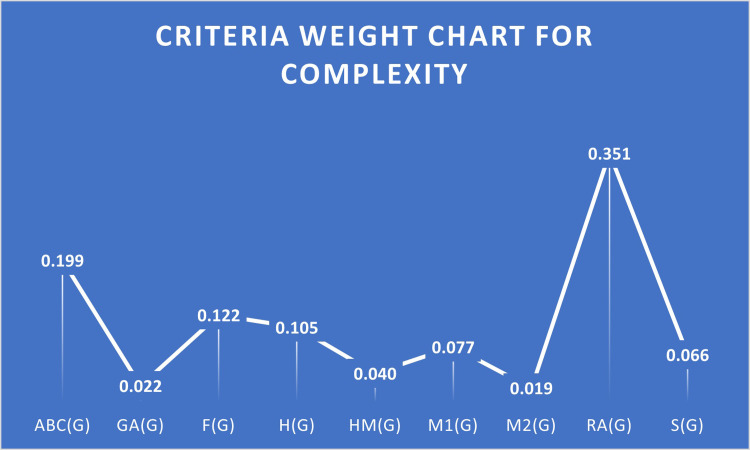
Criteria weight chart for complexity.

A higher correlation among some criteria suggests that this criterion is beneficial. S3 Table in S1 File lists the criteria that are beneficial and non-beneficial for Complexity case. The decision matrix will be normalized using beneficial criteria with the linear max technique. Pairwise Comparison of criteria under Complexity is given in Table 10, Normalized Decision Matrix for Complexity Case is given in Table 11, and Ranks Of HIV drugs for Complexity Case are given in Table 12. Fig 4 provides the criteria weight chart for Complexity.

**Table 10:** Pairwise comparison matrix for complexity case.

	ABC(ℳ)	GA(ℳ)	F(ℳ)	H(ℳ)	HM(ℳ)	M1(ℳ)	M2(ℳ)	RA(ℳ)	S(ℳ)
**ABC(**ℳ**)**	1.000	7.000	3.000	3.000	5.000	3.000	7.000	0.333	3.000
**GA(**ℳ**)**	0.143	1.000	0.200	0.200	0.333	0.200	2.000	0.111	0.200
**F(**ℳ**)**	0.333	5.000	1.000	2.000	3.000	4.000	5.000	0.200	2.000
**H(**ℳ**)**	0.333	5.000	0.500	1.000	3.000	4.000	5.000	0.200	2.000
**HM(**ℳ**)**	0.200	3.000	0.333	0.333	1.000	0.333	3.000	0.143	0.333
**M1(**ℳ**)**	0.333	5.000	0.250	0.250	3.000	1.000	5.000	0.200	4.000
**M2(**ℳ**)**	0.143	0.500	0.200	0.200	0.333	0.200	1.000	0.111	0.200
**RA(**ℳ**)**	3.000	9.000	5.000	5.000	7.000	5.000	9.000	1.000	5.000
**S(**ℳ**)**	0.333	5.000	0.500	0.500	3.000	0.250	5.000	0.200	1.000
**Weights**	0.199	0.022	0.122	0.105	0.040	0.077	0.019	0.351	0.066

The Consistency Ratio for these weights is 0.083975 < 0.1 which means weights are consistent.

**Table 11:** Normalized decision matrix for complexity case.

*DRUG*	ABC(ℳ)	GA(ℳ)	F(ℳ)	H(ℳ)	HM(ℳ)	M1(ℳ)	M2(ℳ)	RA(ℳ)	S(ℳ)
***Vidaza***	1.132	0.342	1.206	0.336	1.200	1.158	1.193	0.335	0.337
**Lamivudine**	1.000	0.307	1.000	0.305	1.000	1.000	1.000	0.300	0.303
**Darunavir**	2.224	0.660	2.407	0.653	2.343	2.250	2.273	0.655	0.659
**Disovey**	1.068	0.324	1.103	0.320	1.097	1.079	1.091	0.317	0.320
**Maraviroc**	2.569	0.785	2.773	0.749	2.751	2.658	2.727	0.738	0.765
**Tenofovir**	2.303	0.679	2.289	0.686	2.222	2.237	2.148	0.686	0.688
**Tripranavir**	2.843	0.850	3.247	0.828	3.135	2.947	3.011	0.826	0.847
**Atazanavir**	3.379	1.000	3.485	1.000	3.378	3.342	3.261	1.000	1.000
**Lopinavir**	3.010	0.924	2.876	0.923	2.886	2.947	2.898	0.904	0.915
**Abacavir**	1.478	0.465	1.577	0.440	1.600	1.553	1.625	0.426	0.446
**Etravirine**	1.772	0.532	1.835	0.516	1.816	1.789	1.795	0.514	0.523
**Nelfinavir**	2.799	0.830	3.113	0.804	3.011	2.868	2.898	0.804	0.817
**Toreforant**	2.346	0.704	2.361	0.699	2.319	2.316	2.273	0.693	0.700

**Table 12:** Ranks Of HIV drugs for complexity case.

*DRUG*	*R*	RANK
***Vidaza***	0.714269	11
**Lamivudine**	0.620598	13
**Darunavir**	1.401822	8
**Disovey**	0.667441	12
**Maraviroc**	1.620461	5
**Tenofovir**	1.411658	7
**Tripranavir**	1.82166	3
**Atazanavir**	2.094578	1
**Lopinavir**	1.841135	2
**Abacavir**	0.935832	10
**Etravirine**	1.100941	9
**Nelfinavir**	1.770702	4
**Toreforant**	1.446603	6

## 4. Conclusion

The thirteen targeted drugs used in HIV have been observed using the very useful MCDM technique known as AHP. The background setting for AHP is highly dependent on the evaluations and has been observed using QSPR modelling by taking three properties into consideration: complexity, boiling point, and flash point. This concept is used in pharmacological studies where the three characteristics have a significant impact on the amount of medications taken. The values we considered from QSPR analysis include the corelation coefficients representing the relationship between each individual property and the targeted degree based topological indices, as well as error values generated during QSPR analysis.

The inclusion of inputs from degree-based chemical indices is justified by their high importance, which highlights each index’s capacity to forecast a certain feature.

We ranked 13 drugs *Vidaza*, Darunavir, Lamivudine, Maraviroc, Tripranavir, Atazanavir, Tenofovir, Toreforant, Abacavir, Nelfinavir, and Etravirine used to cure HIV using the multicriteria decision-making process AHP based on three characteristics: complexity, boiling point, and flash point. We find that Atazanavir ranks top, followed by Lopinavir (rank 2) and Tripranavir (rank 3), Nelfinavir (rank4), Maraviroc (rank5), Toreforant (rank6), Tenofovir(rank7), Darunavir(rank 8), Etravirine (rank9), Abacavir (rank10), (rank11), Disovey(rank12), and Lamivudine(rank13) based on the three properties studied: boiling point, flash point, and complexity.

**Fig 5:**
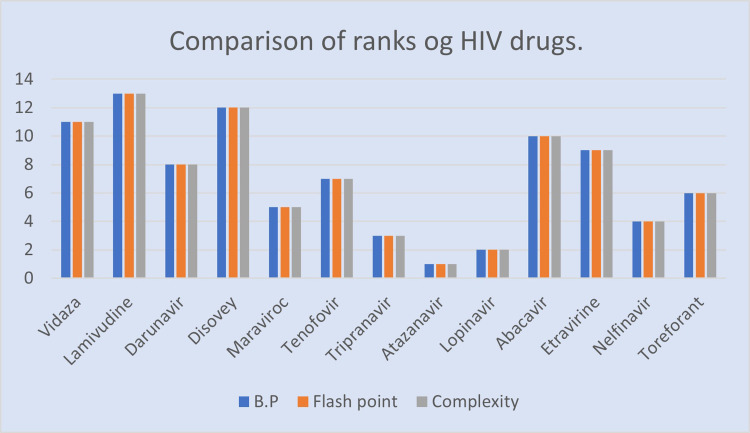
Comparison of ranks.

Fig 5 can be used to display rank comparisons. Furthermore, this perspective ushers in a new era that will benefit ranking structures in a variety of disciplines, including chemistry, biology, and physics. When chemical indices play an important role in QSPR models, we can identify targeted features and see how these evaluations influence the rank ordering of various structures based on diverse criteria. It is vital to highlight that the emphasis is not only on the chemical indices themselves, but also on the chemical cost associated with each desired index in creating the best structure. The current research outlines an effective method for selecting and analysing the best chemical compounds for drugs development, as well as producing unique, modern pharmaceuticals. The purpose is to move over QSPR and investigate how to combine multiple drugs that are used to treat HIV. Applying these methods, we hope to determine the optimal drug combinations for more potent HIV treatments.

## Supporting information

S1 File The supporting information file that is connected to the manuscript contains more supporting data, such as supporting tables and figures. (PDF)
