# Supplementary material for: Optimization of HIV drugs through MCDM technique Analytic Hierarchy Process(AHP)
Source: PLoS One. 2025 Jan 17;20(1):e0316617. doi: 10.1371/journal.pone.0316617 (PMC11741607; doi:10.1371/journal.pone.0316617)
Supplement: S1 File — (PDF) [file pone.0316617.s001.pdf]

Optimization of HIV drugs through MCDM technique Analytic Hierarchy Process(AHP)

Fozia Bashir Farooq[1], Sobia Sultana[1] , Nouf Abdulrahman Alqahtani [1], Muhammad Imran[2]

1. Department of Mathematics and Statistics, College of Science, Imam Mohammad Ibn Saud Islamic University (IMSIU), Saudi Arabia.

2. Prince Mohammad Bin Fahd University, P.O. Box 1664, Al Khobar 31952, Saudi Arabia.

P.O. Box 15551, Al Ain, United Arab Emirates.

e-mail: fozia.gc@gmail.com, ffarooq@imamu.edu.sa

Table S1: Beneficial Criteria for Boiling point case.

Correlation Coefficient	0.865	0.882	0.813	o.877	0.835	0.860	0.859	0.868	0.876
DRUG	ABC($\mathcal{M}$)	GA($\mathcal{M}$)	F($\mathcal{M}$)	H($\mathcal{M}$)	HM($\mathcal{M}$)	M1($\mathcal{M}$)	M2($\mathcal{M}$)	RA($\mathcal{M}$)	S($\mathcal{M}$)
Vidaza	12.96	17.265	234	7.63	444	88	105	8.04	8.25
Lamivudine	11.45	15.49	194	6.93	370	76	88	7.2	7.42
Darunavir	25.46	33.33	467	14.86	867	171	200	15.72	16.13
Disovey	12.23	16.36	214	7.27	406	82	96	7.61	7.83
Maraviroc	29.42	39.63	538	17.03	1018	202	240	17.72	18.71
Tenofovir	26.37	34.29	444	15.6	822	170	189	16.47	16.83
Tripranavir	32.55	42.93	630	18.84	1160	224	265	19.83	20.72
Atazanavir	38.69	50.51	676	22.74	1250	254	287	24.01	24.46
Lopinavir	34.46	46.66	558	21	1068	224	255	21.71	22.39
Abacavir	16.92	23.51	306	10	592	118	143	10.24	10.92
Etravirine	20.29	26.87	356	11.73	672	136	158	12.33	12.79
Nelfinavir	32.05	41.92	604	18.28	1114	218	255	19.3	19.98
Toreforant	26.86	35.55	458	15.9	858	176	200	16.65	17.11
B+	11.45	50.51	194	22.74	370	76	88	24.01	24.46

Table S2: Beneficial Criteria for flash point case.

Correlation Coefficient	0.864	0.882	0.813	o.877	0.835	0.860	0.859	0.868	0.876
DRUG	ABC($\mathcal{M}$)	GA($\mathcal{M}$)	F($\mathcal{M}$)	H($\mathcal{M}$)	HM($\mathcal{M}$)	M1($\mathcal{M}$)	M2($\mathcal{M}$)	RA($\mathcal{M}$)	S($\mathcal{M}$)
Vidaza	12.96	17.265	234	7.63	444	88	105	8.04	8.25
Lamivudine	11.45	15.49	194	6.93	370	76	88	7.2	7.42
Darunavir	25.46	33.33	467	14.86	867	171	200	15.72	16.13
Disovey	12.23	16.36	214	7.27	406	82	96	7.61	7.83
Maraviroc	29.42	39.63	538	17.03	1018	202	240	17.72	18.71
Tenofovir	26.37	34.29	444	15.6	822	170	189	16.47	16.83
Tripranavir	32.55	42.93	630	18.84	1160	224	265	19.83	20.72
Atazanavir	38.69	50.51	676	22.74	1250	254	287	24.01	24.46

Lopinavir	34.46	46.66	558	21	1068	224	255	21.71	22.39
Abacavir	16.92	23.51	306	10	592	118	143	10.24	10.92
Etravirine	20.29	26.87	356	11.73	672	136	158	12.33	12.79
Nelfinavir	32.05	41.92	604	18.28	1114	218	255	19.3	19.98
Toreforant	26.86	35.55	458	15.9	858	176	200	16.65	17.11
B+	11.45	50.51	194	22.74	370	76	88	24.01	24.46

Table S3: Beneficial Criteria for Complexity case.

Correlation Coefficient.	0.926	0.919	0.923	0.923	0.922	0.924	0.919	0.986	0.923
DRUG	ABC($\mathcal{M}$)	GA($\mathcal{M}$)	F($\mathcal{M}$)	H($\mathcal{M}$)	HM($\mathcal{M}$)	M1($\mathcal{M}$)	M2($\mathcal{M}$)	RA($\mathcal{M}$)	S($\mathcal{M}$)
Vidaza	12.96	17.265	234	7.63	444	88	105	8.04	8.25
Lamivudine	11.45	15.49	194	6.93	370	76	88	7.2	7.42
Darunavir	25.46	33.33	467	14.86	867	171	200	15.72	16.13
Disovey	12.23	16.36	214	7.27	406	82	96	7.61	7.83
Maraviroc	29.42	39.63	538	17.03	1018	202	240	17.72	18.71
Tenofovir	26.37	34.29	444	15.6	822	170	189	16.47	16.83
Tripranavir	32.55	42.93	630	18.84	1160	224	265	19.83	20.72
Atazanavir	38.69	50.51	676	22.74	1250	254	287	24.01	24.46
Lopinavir	34.46	46.66	558	21	1068	224	255	21.71	22.39
Abacavir	16.92	23.51	306	10	592	118	143	10.24	10.92
Etravirine	20.29	26.87	356	11.73	672	136	158	12.33	12.79
Nelfinavir	32.05	41.92	604	18.28	1114	218	255	19.3	19.98
Toreforant	26.86	35.55	458	15.9	858	176	200	16.65	17.11
B+	38.69	15.49	194	6.93	370	254	88	24.01	7.42

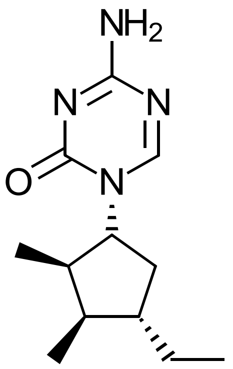

(a) *Vidaza*

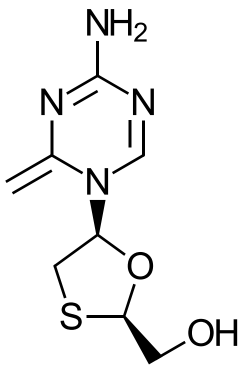

(b) Lamivudine

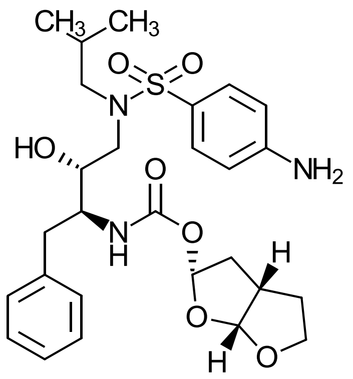

(c) Darunavir

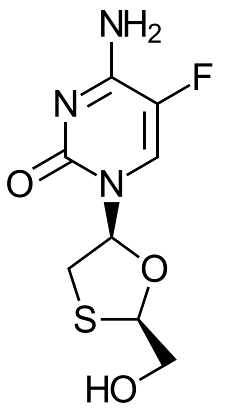

(d) Disovey

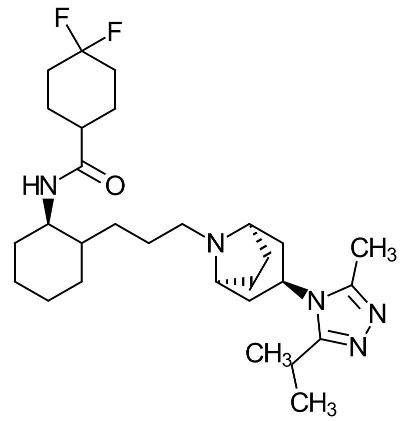

(e) Maraviroc

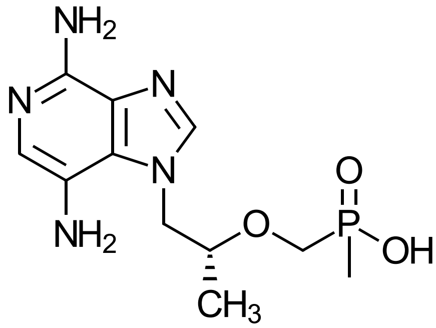

(f) Tenofovir

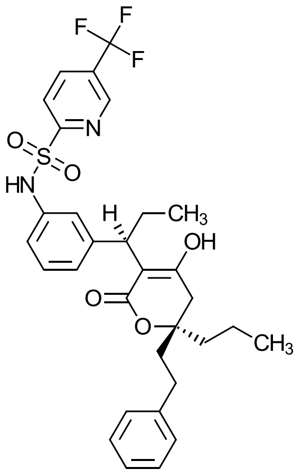

(g) Tipranavir

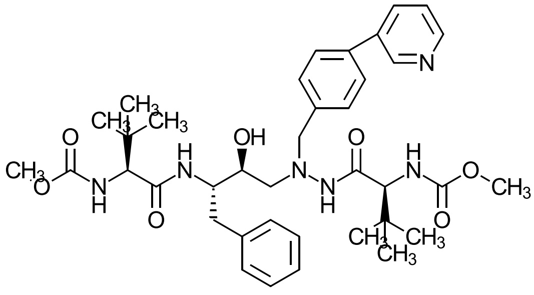

(h) Atazanavir

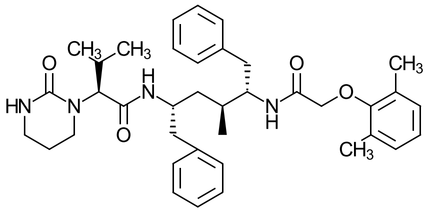

(i) Lopinavir

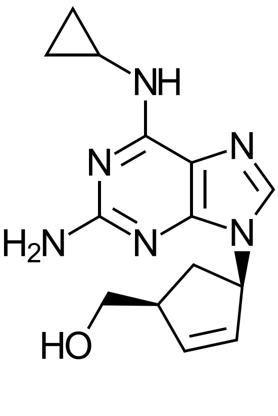

(a) Abacavir

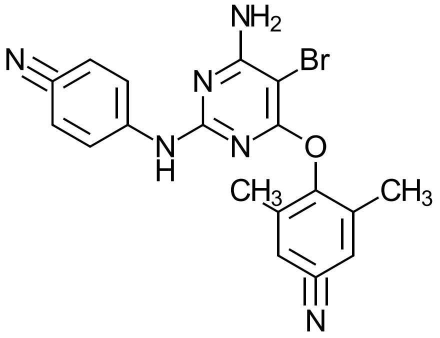

(b) Etravirine

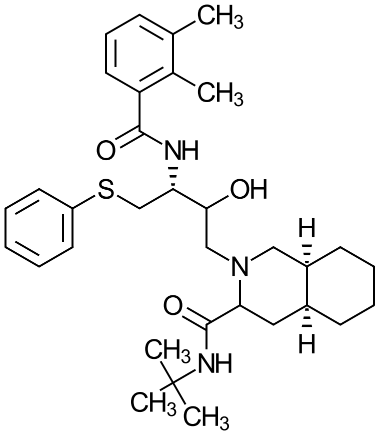

(c) Nelfinavir

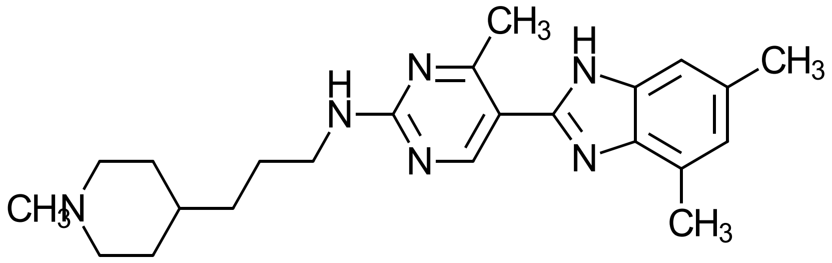

(e) Maraviroc

Figure S1. Molecular structure of HIV drugs
